# Superconductivity in Nanosystems: A Fruitful Path to New Phenomenology in Quantum Materials

**DOI:** 10.3390/nano13030592

**Published:** 2023-02-01

**Authors:** Manuel V. Ramallo

**Affiliations:** 1 Quantum Materials and Photonics Research Group (QMatterPhotonics), Department of Particle Physics, University of Santiago de Compostela, 15782 Santiago de Compostela, Spain; ramallo@cond-mat.eu or mv.ramallo@usc.es; 2 Instituto de Materiais (iMATUS), University of Santiago de Compostela, 15782 Santiago de Compostela, Spain

In the recent years, the landscape of the research in superconductivity has experienced a progressive focus on varied superconducting systems, which share as common primary characteristics the reduction of some of their dimensionalities and the emergence of qualitatively novel phenomenology with respect to bulk superconducting materials.

This includes nanosystems, such as, e.g., very thin films [1], tunnel–junction-based hybrid devices [2], superconductors with nanospaced defects or vortex pinning centers [3], interfacial superconductivity [4], etc.

Superconductivity is a collective phenomenon in which spatial correlations and coherences are crucial. It is not surprising, then, that the effects of reduced dimensionality may affect the superconducting properties. Correspondingly, the nanosizing and/or nanostructuring of superconductors often leads to the advent of novel phenomenology not present in bulks and which is also qualitatively different from the one in other nanosystems due to the unique quantum properties of superconductors. The result is that the study of superconductivity in nanosystems is uncovering rich, singular, and qualitatively novel phenomenologies, often quantum in character. The interest of these discoveries is two-fold. On the one hand, their study is academically sound per se. On the other hand, their study is relevant to various applications where superconductors are preferred materials. These phenomena are exemplified in the fields of quantum computing and communications, high magnetic field applications, quantum sensorics, etc. [5]

Well aware of the growing interlinks between nanoscience and superconductivity, the journal *Nanomaterials* in this Special Issue *“Superconductivity in Nanosystems”* gathers various articles presenting original research in the topic, including the fabrication of new systems, the experimental observation of new phenomenologies in them, and/or the explanation of the latter in terms of theory models.

Often, the new properties result from the interaction of the intrinsic superconducting order (superconducting wave function, quantized vortices, etc.) and an additional, nanoengineered order. The first has a tendency to organize itself following characteristic lengths, such as the superconducting coherence length or the distance between vortices. Those lengths can be targeted by nanostructuring.

In the following paragraphs, we very briefly present the articles included in this Special Issue by crudely grouping them by the physical orders whose competitions may be seen as the main cause of the reported new phenomenology in each case. Hopefully, this will serve not only to highlight these interesting works, but also to emphasize what I believe is a general feeling in our research community: that, in the field of superconductivity in nanosystems, abundant new phenomenology still awaits to be discovered as different forms of order competitions and nanogeometries are explored.

Firstly, two articles in this Special Issue are dedicated to some interesting interrelations between magnetic and superconducting orders that become realizable through nanoscience. Winarsih et al. [6] conducted studies of nanoparticles of the La ${}_{2-x}$ Sr ${}_{x}$ CuO ${}_{4}$ cuprate superconductor, observing that a reduction of the particle size down to the nanometric scale is accompanied by the appearance of magnetic correlation in the Cu spin fluctuations. They likewise observed a change in the bond distance between Cu and O ions in the conducting layer, which seems to correlate with that new magnetism.

Also, Vettoliere et al. [7] focused their attention on the case of Josephson tunnel nanojunctions, whereto a ferromagnetic element is added. They report the fabrication of such systems using multistage deposition–lithography processes. The so-obtained junctions become switchable using the influence of the magnetic element over the superconducting wave function, which may be of great relevance in the area of the emerging quantum computing and communications technologies [5,7]. The junctions are Al-based and bear good prospects of integrability in different types of quantum circuits.

Two articles in this Special Issue explore aspects related to the increase, by means of nanoengineering, of pinning centers for the superconducting quantum vortices. It is today well known that such pinning may increase the critical current, $j_{c}$, which is of crucial importance, e.g., for high-field applications [3,5,8]. In their article, Ivan et al. [9] conduct studies to demonstrate, among other aspects, that the frequency-dependent $j_{c}$ of their samples with self-assembled defects can be explained by using a new and simple, but effective, method involving AC susceptibility measurements. It is a useful development that also points to implications concerning the dependence of the pinning potential with the probing current.

The article by Backmeister et al. [10] is another deft example of the richness of new possible phenomenologies obtainable through nanostructured pinning. This research reports the observation of an ‘ordered Bose glass state’ for the superconducting vortex lines. For that, a superconducting system with a regular and hexagonal array of pinning centers is manufactured, with only 30 nm spacing between them, which allows for the entanglement of vortices.

Finally, this Special Issue also includes two articles that exemplify how new phenomenology can appear from the frustration of the characteristic lengths of the superconducting wave function by the finite sizes of the system. In particular, Adhikari et al. [11] measured the resistive superconducting transition in Fe-implanted NbN nanometric-thin films, observing the appearance of up to six different regimes in the vicinity of that transition. They managed to interpret this rich phenomenology in terms of a finite-size granular model of the material, and the corresponding considerations on the bosonic and fermionic electrical conduction channels that are expected to coexist near the transition. The different regimes appear as a result of the progressive formation of local conduction clusters, as well as eventually as a result of percolation paths between them.

The article by Botana et al. [12] explores the precursor superconductor state as the resistive transition is approached, in this case in very thin films of cuprate superconductors composed of only a few layers of unit cells. It is known that measurements in such films are not satisfactorily reproduced by the critical phenomena equations for bulk samples, nor for single two-dimensional plane superconductors [13,14]. Botana et al. [12] presented model calculations for the precursor superconducting wave funtion, which are specifically adapted to the few-layer case, obtaining good agreement with the data.

Of course, a single Special Issue cannot fully cover the entire broad spectrum of research aspects being currently studied on the frontiers between superconductivity and nanoscience. Hopefully, future additional volumes devoted to this same general topic will serve to partially fill that void, transforming, at the same time, into a forum for the presentation of further original research in this invigorating and auspicious field.

## Data Availability

Not applicable.
